# Crystal structure of 4-nitro­phenyl 6-*O*-ethyl-β-d-galacto­pyran­oside monohydrate

**DOI:** 10.1107/S2056989017004595

**Published:** 2017-03-28

**Authors:** Bruno Leonardo Silva, Ricardo José Alves, Nivaldo Lúcio Speziali

**Affiliations:** a Departamento de Produtos Farmacêuticos – Faculdade de Farmácia – Universidade Federal de Minas Gerais – Avenida Antônio Carlos 6627, Belo Horizonte MG, 31.270-901, Brazil; b Departamento de Física – Instituto de Ciências Exatas – Universidade Federal de Minas Gerais – Avenida Antônio Carlos 6627, Belo Horizonte MG, 31.270-901, Brazil

**Keywords:** crystal structure, d-galactose, nitro­phenyl galacto­pyran­osides, pyran­oid ring, hydrogen bonding

## Abstract

The synthesis and crystal structure of the title compound, C_14_H_19_NO_8_·H_2_O, prepared in three steps from 6-*O*-ethyl-1,2;3,4-di-*O*-iso­propyl­idene-α-d-galacto­pyran­ose using protecting-group strategies employed in carbohydrate chemistry, is reported. The asymmetric unit consists of a single galactoside mol­ecule, in which the pyran­oid ring has a ^4^ *C*_1_ conformation and the 4-nitro­phenyl moiety is essentially planar. In the crystal, each carbohydrate is surrounded by other d-galactose residues and water mol­ecules, linked by O—H⋯O hydrogen bonds involving all hy­droxy groups, giving a two-dimensional substructure lying parallel to (100) and extended into three dimensions by C—H⋯O inter­actions.

## Abstract 2

The pyran­oid ring of the title compound C_14_H_19_NO_8_·H_2_O has a ^4^ *C*_1_ conformation and the 4-nitro­phenyl moiety is essentially planar. The galactoside mol­ecules are connected by several O—H⋯O hydrogen bonds, forming a sheet lying parallel to (100), and by inter­molecular C—H⋯O inter­actions.

## Chemical context   

Small mol­ecules containing d-galactose moieties substituted at non-anomeric positions have been assayed against galacto­sidases (Viana *et al.*, 2011 ▸; McCarter *et al.*, 1992 ▸; Huber & Gaunt, 1983 ▸) and lectins (Butera *et al.*, 2009 ▸; Salameh *et al.*, 2005 ▸). *Trypanosoma cruzi trans*-sialidase (TcTS) (Mendonça-Previato *et al.*, 2010 ▸), an enzyme involved in Chagas’s disease infection, is inhibited by β-d-galacto­pyran­osides substituted at the C6 ring site, which are in general more potent than the corresponding analogues modified at other ring positions of the carbohydrate (Harrison *et al.*, 2011 ▸). In this context, the title compound C_14_H_19_NO_8_ was designed and synthesized to be evaluated against TcTS and *T. cruzi* invasion of host cells. The synthesis and crystal structure of this compound as the monohydrate (I) is reported herein.
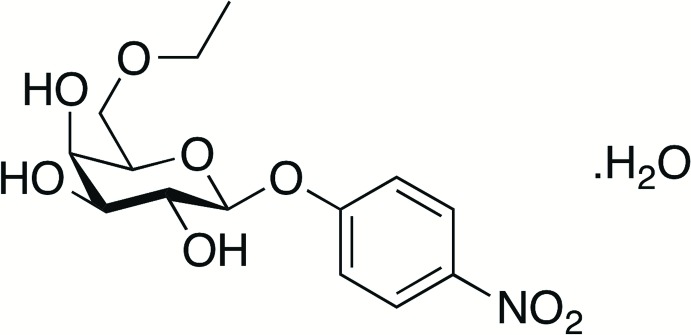


## Structural commentary   

**Figure 1:**
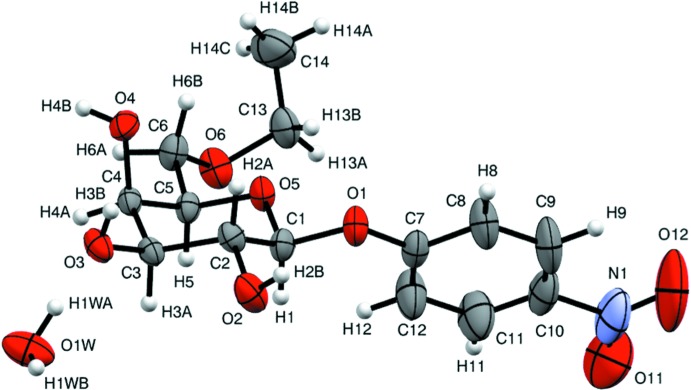
The mol­ecular structure of the title compound with the atom labelling. Displacement ellipsoids are drawn at the 50% probability level.

In the structure of the title monohydrated compound (I) (Fig. 1 ▸), the pyran­oid ring adopts a ^4^ *C*_1_ conformation, with puckering parameters *Q* = 0.569 (2) Å, θ = 4.6 (2)° and φ = 51 (3)°. The anomeric *beta* form and d-galacto configuration of the carbohydrate with C1(*S*), C2(*R*), C3(*S*), C4(*R*) and C5(*R*) are consistent with that expected from the synthesis. The length of the glycosidic bond is 1.408 (2) Å and the bond angles around the anomeric carbon atom (C1) range from 106.40 (16) to 111.35 (17)°. The 4-nitro­phenyl substituent at C1 located in an equatorial position is essentially planar, with a r.m.s. deviation of 0.02 Å for non-hydrogen atoms [torsion angle C9—C10—N1—O11 = 179.0 (4)°]. The angle between the mean plane of the 4-nitro­phenyl substituent (defined by atoms C7–C12/N1/O11/O12) and the mean sugar plane (defined by C1–C5/O5 atoms) is 57.45 (11)° [torsion angle O5—C1—O1—O7 = −79.8 (2)°]. An intra­molecular C13—H13*B*⋯O5 inter­action is also present (Table 1 ▸).

## Supra­molecular features   

**Figure 2:**
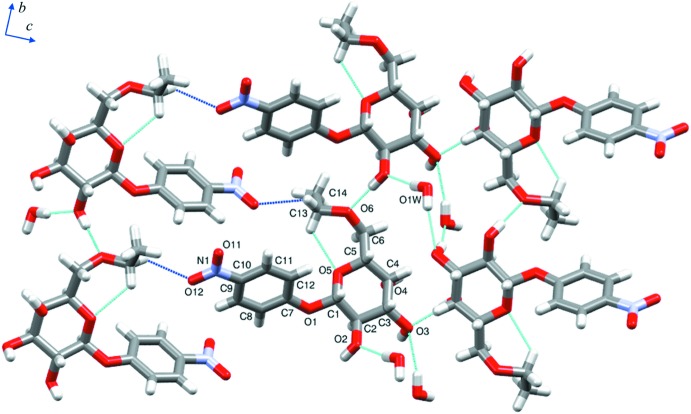
Selected inter­actions in the crystal lattice with O—H⋯O hydrogen bonds shown as turquoise dashed lines and C—H⋯O inter­actions shown as blue dashed lines.

**Figure 3:**
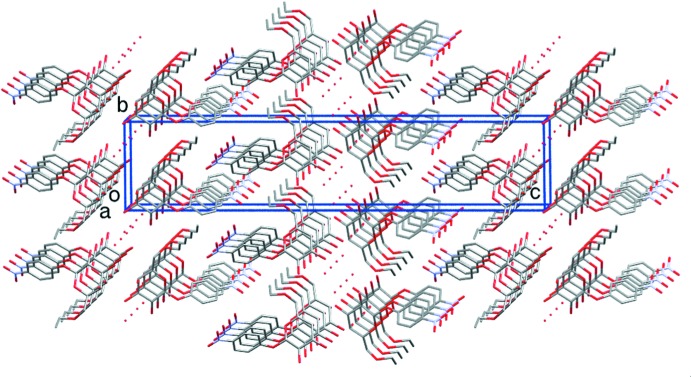
Crystal packing of the title compound, showing the stacked galactoside mol­ecules along the *a* axis. For clarity, H atoms are not shown.

In the crystal, a carbohydrate moiety is connected to eight neighboring d-galactose residues by several direct and water-mediated classical hydrogen bonds (Table 1 ▸), establishing a network of inter­actions (Fig. 2 ▸). Regarding only the O—H⋯O inter­action type, there are O2—H2*B*⋯O6^i^, O3—H3*B*⋯O1*W* ^ii^ and O4—H4*B*⋯O3^iii^ hydrogen bonds. In addition, there is a single-water bridge connecting O3 to O2 of a nearby galactoside mol­ecule (O1*W*—H1*WA*⋯O2^iv^ and O1*W*—H1*WB*⋯O3^v^; for symmetry codes, see Table 1 ▸). A two-dimensional substructure in the form of a sheet lying parallel to (100) is formed. The overall three-dimensional supra­molecular aggregation is completed by inter­molecular C—H⋯O inter­actions: C3—H3*A*⋯O4^vi^ connects carbohydrate rings stacked along the *a* axis and C13—H13*A*⋯O12^vii^ connects ethyl and nitro groups along the *c* axis. The 4-nitro­phenyl substituent groups are arranged in parallel planes (Fig. 3 ▸), with an inter­planar distance of 3.4355 (14) Å, but the slip angle (48.3°) prevents overlapping and therefore no π–π inter­actions are present [ring-centroid separation = 5.163 (2) Å].

## Database survey   

To the best of our knowledge, this is the first report of the crystal structure of an aryl 6-*O*-substituted-β-d-galacto­pyran­oside in the literature. In the Cambridge Structural Database (Version 5.38; Groom *et al.*, 2016 ▸), the structural data for the closely related analogue 4-nitro­phenyl β-d-galacto­pyran­oside have been deposited (CSD Refcode VUCYO1; Gubica *et al.*, 2009 ▸). Both galactosides are monohydrates and their mol­ecular geometry and inter­molecular inter­action profiles in the crystal lattice are quite similar. The aromatic ring of the 6-unsubstituted galactoside is less planar due to the increased rotation of the N1—C10 bond, since the angle between the mean planes of the phenyl and nitro groups is *ca* 5.1°, compared to 2.6 (5)° in the title compound. According to the authors (Gubica *et al.*, 2009 ▸), the deviation from coplanarity of these fragments in the 4-nitro­phenyl β-d-galacto­pyran­oside structure is due to inter­molecular inter­actions involving the nitro group. In our crystallographic study on compound (I) we did not observe classical hydrogen bonds to 4-nitro­phenyl O-atom acceptors, but only the weak C13—H13*A*⋯O12 inter­action noted above.

## Synthesis and crystallization   

**Figure 4:**
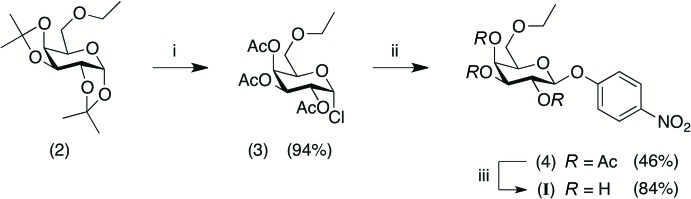
Synthesis of the title compound (I): (i) AcCl, MeOH, HCl_(aq)_, room temperature, closed system; (ii) 4-nitro­phenol, LiOH·H_2_O, acetone/H_2_O, room temperature; (iii) MeONa/MeOH, CH_2_Cl_2_, 273 K. The water mol­ecule of crystallization of (I) is not represented.

The chemical synthesis of 4-nitro­phenyl 6-*O*-ethyl-β-d-galacto­pyran­oside monohydrate (I) was achieved in three steps, as shown in Fig. 4 ▸.

Initially the *O*-alkyl­ation of 1,2;3,4-di-*O*-iso­propyl­idene-α-d-galacto­pyran­ose was carried out as reported in the literature furnishing the 6-*O*-alkyl­ated derivative (**2**) (Cironi & Varela, 2001 ▸; McKeown & Hayward, 1960 ▸). Next, the peracetyl­ated α-d-galacto­pyranosyl chloride (**3**) was prepared in a three-step one-pot reaction as follows. To a solution of (**2**) (0.59 g, 2.06 mmol) in acetyl chloride (2.92 mL, 41.13 mmol) was added methanol (0.42 mL) under ice-bath conditions. The mixture was stirred at room temperature for 2h in a closed system and concentrated hydro­chloric acid (0.34 mL) was then added and the resulting mixture was stirred at room temperature for 24 h, also in a closed system. The reaction was quenched with crushed ice (about 30 mL) and the mixture was extracted with di­chloro­methane (3 × 25 mL). The organic layers were washed with a saturated aqueous sodium bicarbonate solution (2 × 60 mL) and water (60 mL), then dried over anhydrous sodium sulfate and concentrated. The brown oil obtained (0.68 g, 94% yield) was used in the next step without further purification.

Classical procedures in carbohydrate chemistry were employed in the next two steps (Conchie *et al.*, 1957 ▸). The glycosyl­ation of 4-nitro­phenol with (**3**) in alkaline medium gave (**4**) in 46% yield. Treatment with sodium methoxide to remove the acetyl groups furnished (I) (as the monohydrate), in 84% yield. Colorless crystals of (I) (m.p. 424.1–424.9 K) suitable for X-ray diffraction analysis were obtained by slow evaporation of an acetone solution (about 0.7 mg/mL) at room temperature.

Spectrometric data. [α]_D_^28^ −46 (*c* 1.0, DMSO). IR 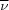
_max_ (cm^−1^): 3354 (O—H), 1608, 1592, 1493 (C=C), 1511, 1349 (NO_2_), 1249, 1074 (C—O), 846 (C—H aromatic out-of-plane bending). ^1^H NMR (400 MHz, DMSO-*d_6_*): *δ*_H_ 8.20 (*d*, 2H, *J*_ortho_ 9.2 Hz, *CH*CNO_2_), 7.22 (*d*, 2H, *J*_ortho_ 9.2 Hz, OC*CH*), 5.28 (*d*, 1H, *J*_OH-2,2_ 5.2 Hz, OH-2), 5.05 (*d*, 1H, *J*_1,2_ 7.6 Hz, H-1), 4.91 (*d*, 1H, *J*_OH-3,3_ 5.7 Hz, OH-3), 4.64 (*d*, 1H, *J*_OH-4,4_ 4.6 Hz, OH-4), 3.85 (*t*, 1H, *J*_5,6a_ 5.4 Hz, *J*_5,6b_ 5.4 Hz, H-5), 3.71–3.66 (*m*, 1H, H-4), 3.63 (*ddd*, 1H, *J*_2,1_ 7.6 Hz, *J*_2,OH-2_ 5.2 Hz, *J*_2,3_ 9.2 Hz, H-2), 3.55 (*dd*, 1H, *J*_6a,5_ 5.4 Hz, *J*_6a,6b_ 10.2 Hz, H-6a), 3.50–3.39 (*m*, 4H, H-3, H-6b and O*CH_2_*CH_3_), 1.09 (*t*, 3H, *J*_ortho_ 6.9 Hz, OCH_2_ *CH_3_*). ^13^C NMR (100 MHz, DMSO-*d_6_*): *δ*_C_ 162.4 (O*C*CH), 141.6 (CH*C*NO_2_), 125.6 (*CH*CNO_2_), 116.5 (OC*CH*), 100.3 (C-1), 73.8 (C-5), 73.0 (C-3), 70.0 (C-2), 69.1 (C-6), 68.3 (C-4), 65.7 (O*CH_2_*CH_3_), 15.1 (OCH_2_ *CH_3_*).

## Refinement   

Crystal data, data collection and structure refinement details are summarized in Table 2 ▸. Oxygen-bound H atoms were located in a difference-Fourier map and refined with distance restraints of 0.82 Å (hy­droxy group H) and 0.89 Å (water H) with *U*_iso_(H) = 1.5 *U*_eq_(O). Carbon-bound H atoms were constrained to an ideal geometry with C—H distances in the range 0.93–0.98 Å, *U*_iso_(H) = 1.5 *U*_eq_(C) for methyl H atoms and *U*_iso_(H) = 1.2 *U*_eq_(C) for other H atoms. In the absence of significant anomalous scattering effects, the Flack structure parameter (Flack, 1983 ▸) is essentially meaningless in this analysis and the absolute configuration is inferred from the known d-galacto configuration of the starting material, and remained unchanged during the synthesis. The *beta* configuration of C1 is confirmed by the coupling constant *J*_1,2_ = 7.6 Hz, obtained from NMR spectroscopy.

## Supplementary Material

Crystal structure: contains datablock(s) I. DOI: 10.1107/S2056989017004595/zs2376sup1.cif

CCDC reference: 1539718

Additional supporting information:  crystallographic information; 3D view; checkCIF report

## Figures and Tables

**Table 1:** Hydrogen-bond geometry (Å, °)

*D*—H⋯*A*	*D*—H	H⋯*A*	*D*⋯*A*	*D*—H⋯*A*
O2—H2*B*⋯O6^i^	0.82	1.85	2.670 (2)	179
O3—H3*B*⋯O1*W* ^ii^	0.82	1.91	2.711 (2)	167
O4—H4*B*⋯O3^iii^	0.82	1.97	2.785 (2)	170
O1*W*—H1*WA*⋯O2^iv^	0.89	1.90	2.776 (3)	166
O1*W*—H1*WB*⋯O3^v^	0.89	2.39	3.208 (2)	154
C3—H3*A*⋯O4^vi^	0.98	2.35	3.283 (3)	158
C13—H13*A*⋯O12^vii^	0.97	2.55	3.167 (3)	121
C13—H13*B*⋯O5	0.97	2.53	3.173 (3)	123

Symmetry codes: (i) 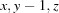; (ii) 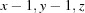; (iii) 
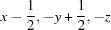
; (iv) 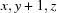; (v) 
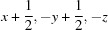
; (vi) 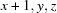; (vii) 
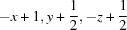
.

**Table 2:** Experimental details

Crystal data	
Chemical formula	C_14_H_19_NO_8_·H_2_O
*M*_r_	347.32
Crystal system, space group	Orthorhombic, *P*2_1_2_1_2_1_
Temperature (K)	293
*a*, *b*, *c* (Å)	5.1628 (3), 8.1593 (3), 38.5755 (16)
*V* (Å^3^)	1624.99 (13)
*Z*	4
Radiation type	Mo *K*α
μ (mm^−1^)	0.12
Crystal size (mm)	0.20 × 0.15 × 0.10

Data collection	
Diffractometer	Rigaku OD Xcalibur, Atlas, Gemini Ultra
Absorption correction	Multi-scan (*CrysAlis PRO*; Rigaku OD, 2015 ▸)
*T*_min_, *T*_max_	0.835, 1.000
No. of measured, independent and observed [*I* > 2/s(*I*)] reflections	26976, 4265, 3597
*R*_int_	0.038
(sin θ/λ)_max_ (Å^−1^)	0.704

Refinement	
*R*[*F* ^2^ > 2σ(*F* ^2^)], *wR*(*F* ^2^), *S*	0.046, 0.108, 1.08
No. of reflections	4265
No. of parameters	218
H-atom treatment	H-atom parameters constrained
Δρ_max_, Δρ_min_ (e Å^−3^)	0.20, −0.23

Computer programs: *CrysAlis PRO* (Rigaku OD, 2015 ▸), *SHELXS97* and *SHELXTL* (Sheldrick, 2008 ▸), *SHELXL2016* (Sheldrick, 2015 ▸), *Mercury* (Macrae *et al.*, 2008 ▸), *PLATON* (Spek, 2009 ▸) and *publCIF* (Westrip, 2010 ▸).

## supplementary crystallographic information

### Crystal data

**Table:**

C_14_H_19_NO_8_·H_2_O	*D*_x_ = 1.420 Mg m^−^^3^
*M**_r_* = 347.32	Melting point: 424.5 K
Orthorhombic, *P*2_1_2_1_2_1_	Mo *K*α radiation, λ = 0.71073 Å
Hall symbol: P 2ac 2ab	Cell parameters from 8847 reflections
*a* = 5.1628 (3) Å	θ = 2.5–28.5°
*b* = 8.1593 (3) Å	µ = 0.12 mm^−^^1^
*c* = 38.5755 (16) Å	*T* = 293 K
*V* = 1624.99 (13) Å^3^	Rod, colourless
*Z* = 4	0.20 × 0.15 × 0.10 mm
*F*(000) = 736	

### Data collection

**Table:**

Rigaku OD Xcalibur, Atlas, Gemini Ultra diffractometer	4265 independent reflections
Radiation source: fine-focus sealed X-ray tube, Enhance (Mo) X-ray Source	3597 reflections with *I* > 2/s(*I*)
Graphite monochromator	*R*_int_ = 0.038
Detector resolution: 10.4186 pixels mm^-1^	θ_max_ = 30.0°, θ_min_ = 2.5°
ω scans	*h* = −7→7
Absorption correction: multi-scan (CrysAlis PRO; Rigaku OD, 2015)	*k* = −11→11
*T*_min_ = 0.835, *T*_max_ = 1.000	*l* = −54→52
26976 measured reflections	

### Refinement

**Table:**

Refinement on *F*^2^	Primary atom site location: structure-invariant direct methods
Least-squares matrix: full	Secondary atom site location: difference Fourier map
*R*[*F*^2^ > 2σ(*F*^2^)] = 0.046	Hydrogen site location: inferred from neighbouring sites
*wR*(*F*^2^) = 0.108	H-atom parameters constrained
*S* = 1.08	*w* = 1/[σ^2^(*F*_o_^2^) + (0.0448*P*)^2^ + 0.4091*P*] where *P* = (*F*_o_^2^ + 2*F*_c_^2^)/3
4265 reflections	(Δ/σ)_max_ < 0.001
218 parameters	Δρ_max_ = 0.20 e Å^−^^3^
0 restraints	Δρ_min_ = −0.23 e Å^−^^3^

### Special details

**Table:**

Geometry. All esds (except the esd in the dihedral angle between two l.s. planes) are estimated using the full covariance matrix. The cell esds are taken into account individually in the estimation of esds in distances, angles and torsion angles; correlations between esds in cell parameters are only used when they are defined by crystal symmetry. An approximate (isotropic) treatment of cell esds is used for estimating esds involving l.s. planes.
Refinement. Refinement of F^2^ against ALL reflections. The weighted R-factor wR and goodness of fit S are based on F^2^, conventional R-factors R are based on F, with F set to zero for negative F^2^. The threshold expression of F^2^ > 2sigma(F^2^) is used only for calculating R-factors(gt) etc. and is not relevant to the choice of reflections for refinement. R-factors based on F^2^ are statistically about twice as large as those based on F, and R- factors based on ALL data will be even larger.

### Fractional atomic coordinates and isotropic or equivalent isotropic displacement parameters (Å^2^)

**Table:**

	*x*	*y*	*z*	*U*_iso_*/*U*_eq_	
O1	0.2973 (3)	0.04259 (19)	0.13258 (4)	0.0359 (4)	
O2	0.3964 (4)	−0.11807 (19)	0.06875 (4)	0.0413 (4)	
H2B	0.3333	−0.1933	0.0800	0.062*	
O3	0.1726 (3)	0.0335 (2)	0.00904 (4)	0.0331 (4)	
H3B	0.0444	−0.0206	0.0142	0.050*	
O4	−0.1450 (3)	0.2630 (2)	0.04353 (4)	0.0340 (4)	
H4B	−0.2160	0.3194	0.0287	0.051*	
O5	0.1951 (3)	0.27596 (18)	0.10442 (3)	0.0300 (3)	
O6	0.1929 (4)	0.63541 (19)	0.10524 (4)	0.0388 (4)	
O11	1.0131 (8)	0.2628 (5)	0.25526 (8)	0.1100 (12)	
O12	0.7670 (9)	0.0893 (4)	0.28005 (6)	0.1237 (14)	
N1	0.8375 (8)	0.1651 (4)	0.25473 (7)	0.0748 (10)	
C1	0.3383 (4)	0.1298 (3)	0.10156 (5)	0.0285 (4)	
H1	0.5228	0.1538	0.0984	0.034*	
C2	0.2387 (5)	0.0236 (3)	0.07205 (5)	0.0283 (4)	
H2A	0.0581	−0.0078	0.0762	0.034*	
C3	0.2607 (4)	0.1215 (2)	0.03863 (5)	0.0257 (4)	
H3A	0.4454	0.1433	0.0350	0.031*	
C4	0.1277 (4)	0.2877 (3)	0.04194 (5)	0.0254 (4)	
H4A	0.1685	0.3539	0.0215	0.030*	
C5	0.2224 (4)	0.3771 (2)	0.07412 (5)	0.0266 (4)	
H5	0.4057	0.4049	0.0711	0.032*	
C6	0.0717 (5)	0.5319 (3)	0.08045 (6)	0.0340 (5)	
H6A	0.0534	0.5910	0.0588	0.041*	
H6B	−0.1005	0.5037	0.0886	0.041*	
C7	0.4348 (5)	0.0860 (3)	0.16169 (6)	0.0350 (5)	
C8	0.3682 (7)	−0.0008 (3)	0.19102 (6)	0.0521 (7)	
H8	0.2354	−0.0778	0.1902	0.062*	
C9	0.5004 (8)	0.0274 (4)	0.22159 (7)	0.0629 (9)	
H9	0.4563	−0.0297	0.2416	0.076*	
C10	0.6952 (6)	0.1392 (4)	0.22221 (6)	0.0515 (7)	
C11	0.7581 (8)	0.2284 (5)	0.19380 (7)	0.0651 (9)	
H11	0.8884	0.3069	0.1950	0.078*	
C12	0.6266 (7)	0.2019 (4)	0.16305 (7)	0.0596 (9)	
H12	0.6684	0.2623	0.1434	0.072*	
C13	0.0993 (6)	0.6211 (3)	0.14011 (6)	0.0431 (6)	
H13A	0.2266	0.6673	0.1558	0.052*	
H13B	0.0799	0.5059	0.1458	0.052*	
C14	−0.1538 (7)	0.7057 (5)	0.14554 (9)	0.0696 (10)	
H14A	−0.1984	0.7024	0.1697	0.104*	
H14B	−0.2858	0.6514	0.1323	0.104*	
H14C	−0.1400	0.8177	0.1381	0.104*	
O1W	0.7487 (4)	0.8396 (2)	0.01513 (5)	0.0579 (6)	
H1WA	0.6231	0.8390	0.0309	0.087*	
H1WB	0.7808	0.7339	0.0114	0.087*	

### Atomic displacement parameters (Å^2^)

**Table:**

	*U*^11^	*U*^22^	*U*^33^	*U*^12^	*U*^13^	*U*^23^
O1	0.0504 (10)	0.0340 (8)	0.0232 (7)	−0.0048 (8)	−0.0058 (7)	0.0061 (6)
O2	0.0589 (11)	0.0259 (8)	0.0391 (9)	0.0043 (8)	0.0131 (8)	0.0045 (7)
O3	0.0378 (8)	0.0389 (8)	0.0226 (7)	−0.0109 (7)	0.0029 (6)	−0.0054 (6)
O4	0.0266 (8)	0.0428 (9)	0.0326 (8)	−0.0055 (7)	−0.0060 (7)	0.0056 (7)
O5	0.0428 (9)	0.0267 (7)	0.0205 (6)	0.0025 (7)	−0.0005 (7)	0.0016 (6)
O6	0.0548 (11)	0.0289 (8)	0.0326 (8)	−0.0085 (8)	0.0020 (8)	−0.0043 (7)
O11	0.101 (2)	0.158 (3)	0.0708 (18)	−0.019 (3)	−0.0407 (17)	−0.029 (2)
O12	0.211 (4)	0.120 (2)	0.0396 (12)	−0.013 (3)	−0.0491 (19)	0.0032 (15)
N1	0.104 (3)	0.082 (2)	0.0393 (14)	0.021 (2)	−0.0273 (16)	−0.0178 (14)
C1	0.0346 (11)	0.0263 (10)	0.0247 (9)	−0.0019 (9)	−0.0023 (9)	0.0051 (8)
C2	0.0335 (11)	0.0256 (9)	0.0258 (9)	−0.0022 (9)	0.0035 (9)	0.0010 (8)
C3	0.0283 (10)	0.0289 (10)	0.0199 (8)	−0.0053 (9)	0.0013 (8)	−0.0012 (8)
C4	0.0275 (10)	0.0287 (10)	0.0200 (9)	−0.0053 (8)	−0.0007 (8)	0.0044 (8)
C5	0.0305 (11)	0.0259 (9)	0.0234 (9)	−0.0055 (9)	−0.0031 (8)	0.0034 (8)
C6	0.0435 (13)	0.0289 (10)	0.0296 (10)	0.0001 (10)	−0.0060 (9)	0.0001 (9)
C7	0.0494 (14)	0.0309 (11)	0.0248 (10)	0.0063 (10)	−0.0055 (10)	−0.0004 (9)
C8	0.085 (2)	0.0448 (15)	0.0267 (11)	−0.0106 (15)	−0.0050 (13)	0.0029 (10)
C9	0.109 (3)	0.0573 (17)	0.0229 (11)	−0.002 (2)	−0.0065 (15)	0.0044 (12)
C10	0.071 (2)	0.0533 (16)	0.0296 (12)	0.0133 (16)	−0.0161 (13)	−0.0103 (12)
C11	0.074 (2)	0.079 (2)	0.0422 (15)	−0.024 (2)	−0.0151 (15)	−0.0040 (15)
C12	0.076 (2)	0.069 (2)	0.0341 (13)	−0.0261 (18)	−0.0139 (14)	0.0098 (13)
C13	0.0567 (16)	0.0445 (14)	0.0282 (11)	−0.0012 (13)	0.0004 (11)	−0.0034 (11)
C14	0.0544 (19)	0.097 (3)	0.0570 (18)	0.0062 (19)	0.0048 (15)	−0.0203 (18)
O1W	0.0500 (11)	0.0482 (11)	0.0753 (13)	−0.0145 (10)	0.0207 (11)	−0.0187 (10)

### Geometric parameters (Å, º)

**Table:**

O1—C1	1.408 (2)	C5—H5	0.9800
O1—C7	1.375 (3)	C5—C6	1.504 (3)
O2—H2B	0.8200	C6—H6A	0.9700
O2—C2	1.420 (3)	C6—H6B	0.9700
O3—H3B	0.8201	C7—C8	1.379 (3)
O3—C3	1.423 (2)	C7—C12	1.370 (4)
O4—H4B	0.8199	C8—H8	0.9300
O4—C4	1.424 (3)	C8—C9	1.382 (4)
O5—C1	1.408 (3)	C9—H9	0.9300
O5—C5	1.438 (2)	C9—C10	1.358 (5)
O6—C6	1.421 (3)	C10—C11	1.355 (4)
O6—C13	1.434 (3)	C11—H11	0.9300
O11—N1	1.207 (5)	C11—C12	1.384 (4)
O12—N1	1.212 (4)	C12—H12	0.9300
N1—C10	1.469 (4)	C13—H13A	0.9700
C1—H1	0.9800	C13—H13B	0.9700
C1—C2	1.520 (3)	C13—C14	1.493 (4)
C2—H2A	0.9800	C14—H14A	0.9600
C2—C3	1.521 (3)	C14—H14B	0.9600
C3—H3A	0.9800	C14—H14C	0.9600
C3—C4	1.526 (3)	O1W—H1WA	0.8898
C4—H4A	0.9800	O1W—H1WB	0.8902
C4—C5	1.520 (3)		
			
C7—O1—C1	119.10 (18)	C6—C5—H5	108.9
C2—O2—H2B	109.5	O6—C6—C5	112.37 (18)
C3—O3—H3B	109.5	O6—C6—H6A	109.1
C4—O4—H4B	109.4	O6—C6—H6B	109.1
C1—O5—C5	111.77 (15)	C5—C6—H6A	109.1
C6—O6—C13	115.74 (19)	C5—C6—H6B	109.1
O11—N1—O12	123.3 (3)	H6A—C6—H6B	107.9
O11—N1—C10	119.0 (3)	O1—C7—C8	114.2 (2)
O12—N1—C10	117.7 (4)	C12—C7—O1	125.6 (2)
O1—C1—H1	110.5	C12—C7—C8	120.2 (2)
O1—C1—C2	107.33 (16)	C7—C8—H8	120.3
O5—C1—O1	106.40 (16)	C7—C8—C9	119.5 (3)
O5—C1—H1	110.5	C9—C8—H8	120.3
O5—C1—C2	111.35 (17)	C8—C9—H9	120.2
C2—C1—H1	110.5	C10—C9—C8	119.5 (3)
O2—C2—C1	109.70 (18)	C10—C9—H9	120.2
O2—C2—H2A	110.3	C9—C10—N1	118.8 (3)
O2—C2—C3	107.99 (16)	C11—C10—N1	119.6 (3)
C1—C2—H2A	110.3	C11—C10—C9	121.6 (3)
C1—C2—C3	108.08 (16)	C10—C11—H11	120.3
C3—C2—H2A	110.3	C10—C11—C12	119.4 (3)
O3—C3—C2	113.03 (16)	C12—C11—H11	120.3
O3—C3—H3A	106.8	C7—C12—C11	119.7 (3)
O3—C3—C4	111.82 (17)	C7—C12—H12	120.1
C2—C3—H3A	106.8	C11—C12—H12	120.1
C2—C3—C4	111.25 (16)	O6—C13—H13A	109.0
C4—C3—H3A	106.8	O6—C13—H13B	109.0
O4—C4—C3	108.80 (17)	O6—C13—C14	112.9 (2)
O4—C4—H4A	109.0	H13A—C13—H13B	107.8
O4—C4—C5	110.55 (17)	C14—C13—H13A	109.0
C3—C4—H4A	109.0	C14—C13—H13B	109.0
C5—C4—C3	110.47 (17)	C13—C14—H14A	109.5
C5—C4—H4A	109.0	C13—C14—H14B	109.5
O5—C5—C4	110.93 (16)	C13—C14—H14C	109.5
O5—C5—H5	108.9	H14A—C14—H14B	109.5
O5—C5—C6	107.41 (17)	H14A—C14—H14C	109.5
C4—C5—H5	108.9	H14B—C14—H14C	109.5
C6—C5—C4	111.64 (17)	H1WA—O1W—H1WB	104.0
			
O1—C1—C2—O2	−67.1 (2)	C1—O5—C5—C6	−177.75 (17)
O1—C1—C2—C3	175.37 (17)	C1—C2—C3—O3	179.94 (18)
O1—C7—C8—C9	177.4 (3)	C1—C2—C3—C4	−53.3 (2)
O1—C7—C12—C11	−177.0 (3)	C2—C3—C4—O4	−70.0 (2)
O2—C2—C3—O3	61.3 (2)	C2—C3—C4—C5	51.5 (2)
O2—C2—C3—C4	−171.91 (17)	C3—C4—C5—O5	−53.3 (2)
O3—C3—C4—O4	57.4 (2)	C3—C4—C5—C6	−173.06 (17)
O3—C3—C4—C5	178.97 (16)	C4—C5—C6—O6	−166.46 (18)
O4—C4—C5—O5	67.2 (2)	C5—O5—C1—O1	179.69 (16)
O4—C4—C5—C6	−52.6 (2)	C5—O5—C1—C2	−63.7 (2)
O5—C1—C2—O2	176.81 (16)	C6—O6—C13—C14	−77.1 (3)
O5—C1—C2—C3	59.3 (2)	C7—O1—C1—O5	−79.8 (2)
O5—C5—C6—O6	71.7 (2)	C7—O1—C1—C2	160.93 (19)
O11—N1—C10—C9	179.0 (4)	C7—C8—C9—C10	−0.5 (5)
O11—N1—C10—C11	−2.0 (5)	C8—C7—C12—C11	1.6 (5)
O12—N1—C10—C9	−2.8 (5)	C8—C9—C10—N1	−178.7 (3)
O12—N1—C10—C11	176.2 (4)	C8—C9—C10—C11	2.3 (5)
N1—C10—C11—C12	178.9 (3)	C9—C10—C11—C12	−2.1 (5)
C1—O1—C7—C8	176.3 (2)	C10—C11—C12—C7	0.1 (6)
C1—O1—C7—C12	−4.9 (4)	C12—C7—C8—C9	−1.4 (5)
C1—O5—C5—C4	60.0 (2)	C13—O6—C6—C5	−96.4 (2)

### Hydrogen-bond geometry (Å, º)

**Table:**

*D*—H···*A*	*D*—H	H···*A*	*D*···*A*	*D*—H···*A*
O2—H2*B*···O6^i^	0.82	1.85	2.670 (2)	179
O3—H3*B*···O1*W*^ii^	0.82	1.91	2.711 (2)	167
O4—H4*B*···O3^iii^	0.82	1.97	2.785 (2)	170
O1*W*—H1*WA*···O2^iv^	0.89	1.90	2.776 (3)	166
O1*W*—H1*WB*···O3^v^	0.89	2.39	3.208 (2)	154
C3—H3*A*···O4^vi^	0.98	2.35	3.283 (3)	158
C13—H13*A*···O12^vii^	0.97	2.55	3.167 (3)	121
C13—H13*B*···O5	0.97	2.53	3.173 (3)	123

Symmetry codes: (i) *x*, *y*−1, *z*; (ii) *x*−1, *y*−1, *z*; (iii) *x*−1/2, −*y*+1/2, −*z*; (iv) *x*, *y*+1, *z*; (v) *x*+1/2, −*y*+1/2, −*z*; (vi) *x*+1, *y*, *z*; (vii) −*x*+1, *y*+1/2, −*z*+1/2.
